# 3DPatch: fast 3D structure visualization with residue conservation

**DOI:** 10.1093/bioinformatics/bty464

**Published:** 2018-06-10

**Authors:** David Jakubec, Jiří Vondrášek, Robert D Finn

**Affiliations:** 1 Department of Bioinformatics, Institute of Organic Chemistry and Biochemistry of the Czech Academy of Sciences, Prague 6, 166 10, Czech Republic; 2 Department of Physical and Macromolecular Chemistry, Faculty of Science, Charles University, Prague 2, 128 43, Czech Republic; 3 European Molecular Biology Laboratory, European Bioinformatics Institute (EMBL-EBI), Wellcome Trust Genome Campus, Cambridge, UK

## Abstract

**Summary:**

Amino acid residues showing above background levels of conservation are often indicative of functionally significant regions within a protein. Understanding how the sequence conservation profile relates in space requires projection onto a protein structure, a potentially time-consuming process. 3DPatch is a web application that streamlines this task by automatically generating multiple sequence alignments (where appropriate) and finding structural homologs, presenting the user with a choice of structures matching their query, annotated with residue conservation scores in a matter of seconds.

**Availability and implementation:**

3DPatch is written in JavaScript and is freely available at http://www.skylign.org/3DPatch/. Mozilla Firefox, Google Chrome, and Safari web browsers are supported. Source code is available under MIT license at https://github.com/davidjakubec/3DPatch.

**Supplementary information:**

Supplementary data are available at *Bioinformatics* online.

## 1 Introduction

The degree of evolutionary conservation of amino acid residues is often indicative of their functional significance (e.g. Celniker *et al.*, 2013). Residues critical for protein folding, hydrophobic core stabilization, intermolecular recognition, or enzymatic activity often manifest lower substitution rates compared to the rest of the protein. The level of variability of amino acid residues can be quantitatively expressed by applying information theory based measures, such as entropy or information content, to individual columns in a multiple sequence alignment (MSA) consisting of a family of evolutionarily related sequences (Wheeler *et al.*, 2014).

Here, we present 3DPatch, a client-side web application facilitating the process of residue-level information content calculations, three-dimensional (3D) structure identification and conservation level-based mark-up. 3DPatch operates at interactive speeds and can be used without any prior knowledge of available homologous 3D structures and without having to construct a MSA in advance.

## 2 Description

3DPatch can accept either a single protein sequence or a profile hidden Markov model (HMM) file as an input. An MSA file with size within the limits set by the HMMER web server (Eddy, 1998; Finn *et al.*, 2015) can be entered instead of a profile HMM. When a sequence is entered, 3DPatch connects to the HMMER web server through its application programming interface (API) and performs a *phmmer* search with the query sequence against the UniProt reference proteomes (The UniProt Consortium, 2017) as a target database.

The *phmmer* search yields a set of sequence regions (domains) from the target database similar to the query sequence. The HMMER web server generates a MSA from the set of individual alignments of significant domain hits to the simple profile HMM used in the search. This MSA is then used to build a more realistic profile HMM featuring position-specific parameters reflecting the frequencies of amino acid residues, insertions and deletions observed at individual columns in the MSA. The initial query sequence entered by the user is aligned to this profile HMM using the HMMER suite program *hmmalign* to provide a residue-level mapping between the query sequence and the profile HMM. *hmmalign* is accessed through an API on the HMMER web server. The alignment of the user-entered sequence to the consensus sequence from the profile HMM is shown in the ‘Sequence alignment’ component of 3DPatch.

3DPatch then connects to the HMMER web server again and calls the *hmmsearch* program with the prepared position-specific profile HMM as a query and the Protein Data Bank (PDB, Mir *et al.*, 2018) as a target database. In this way, 3D structures of proteins similar to the original sequence query are identified accurately and quickly by using the power of profile HMMs and speed of the HMMER3.1 implementation.

Concomitant with the *hmmsearch* request, 3DPatch connects to the Skylign web server (Wheeler *et al.*, 2014) through its API and requests calculation of information content at individual positions in the profile HMM built from the *phmmer* search results. The information content values at individual positions in the profile HMM are shown graphically in the ‘HMM information content profile’ component of 3DPatch. If a profile HMM file is used as an input to 3DPatch, the *phmmer* search is not performed and the profile HMM is directly used as an input to *hmmsearch* and Skylign. In this case, there is no user-entered sequence to be shown in the ‘Sequence alignment’ component, and the query profile HMM consensus sequence is presented.

Residue-level mappings between positions in the profile HMM and individual residues in the identified PDB structures are obtained from the domain alignments included in the *hmmsearch* results. Using these mappings and the profile HMM information content profile, 3DPatch can pair an information content value with each 3D structure amino acid residue assigned to a match state in the domain alignment.

**Fig. 1.**
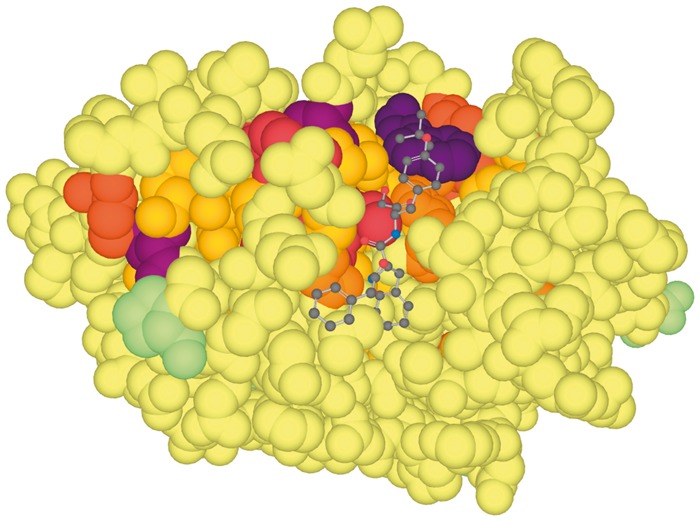
Structure of human cathepsin L1 marked-up with residue information information content in complex with an inhibitor (PDB code 3of8). Darker colors correspond to higher conservation levels; residues outside the alignment region are colored green. Catalytic and binding pocket residues (center top) are clearly distinguished based on the information content. Two conserved cysteine residues forming a disulfide bridge can be seen on the left

In its ‘HMM structure coverage’ component, 3DPatch selects a few informative structures to graphically illustrate which positions in the profile HMM are covered by the identified 3D structures. In addition, a list of all other 3D structures reported by *hmmsearch* is generated. The user can select any of these structures to be visualized with residue-level information content-based mark-up using the LiteMol viewer (Sehnal *et al.*, 2017). The selected structure is automatically downloaded from the Protein Data Bank in Europe (Mir *et al.*, 2018). Figure 1 shows an example of such a visualization. When a structure is selected, the sequence of the marked-up region aligned to the profile HMM consensus sequence will be shown in the ‘Sequence alignment’ component. The user can interact with the aligned sequence using the mouse pointer to see a selected residue in the sequence highlighted in the 3D structure visualization.

3DPatch allows the user to download a save point file once all API queries have been completed. This file can be reloaded into 3DPatch at a later time to restore the application to the saved state.

Supplementary Figure S1 shows a scheme summarizing the entire 3DPatch workflow between the client and federated APIs.

## 3 Discussion

The residue information content displayed by 3DPatch reflects the conservation of the position over the set of sequences included in the MSA. For example, if the MSA contains at some position a highly conserved cysteine residue, but the query sequence or the selected structure contain a serine residue there instead, then the position will still be marked-up as having high information content. In this sense, 3DPatch behaves differently from the popular ConSurf server (Ashkenazy *et al.*, 2016; Celniker *et al.*, 2013). Section ‘Comparison to ConSurf’ in the Supplementary Material provides additional description of significant differences between the two servers. Notably, whereas 3DPatch uses information content as a measure of residue conservation, ConSurf explicitly models evolutionary rate for each position. While the entropy based approach measures overall variability of a site, it does not explicitly account for evolutionary relationships among sequences and the two metrics may yield different results (Echave *et al.*, 2016).

## 4 Conclusion

3DPatch is a web application simplifying the task of calculating protein sequence information content, and projecting conservation level-based mark-up onto protein 3D structures. 3DPatch provides an intuitive user interface that can be embedded into websites wishing to display such information and is compatible with most modern web browsers.

## Supplementary Material

Supplementary Materials
