# Supplementary material for: 3DPatch: fast 3D structure visualization with residue conservation
Source: Bioinformatics. 2018 Jun 10;35(2):332–4. doi: 10.1093/bioinformatics/bty464 (PMC6330005; doi:10.1093/bioinformatics/bty464)
Supplement: Supplementary Materials [file bty464_supplementary_materials.pdf]

Supplementary materials for "3DPatch: fast 3D structure visualization with residue conservation"

David Jakubec, Jiří Vondrášek, Robert D. Finn

Comparison to ConSurf

Compared to 3DPatch, the ConSurf server is a more feature-rich application, presenting the user with more options on how the search for homologous sequences is performed, how the MSA is built, and how are the conservation scores calculated. Besides using different algorithms and target databases to perform these tasks, the ConSurf server notably obtains the residue conservation scores by modelling the sequence evolutionary history based on the MSA results. By contrast, some of what ConSurf performs at the level of the MSA is performed by HMMER when constructing the profile HMM. Notably, HMMER uses a sequence weighting algorithm to down-weight closely related sequences and up-weight distantly related ones, thereby making the model less biased due to uneven phylogenetic representation in the matched sequences. Subsequently, the weights are normalized to sum to a total effective sequence number, which, when using the default entropy-weighting method, reduces the information content per consensus position, thereby emphasising the more conserved positions.

The scores calculated by the ConSurf server represent relative conservation level specific to each sequence. By utilizing the residue entropy-based information content, the conservation level presented by 3DPatch becomes an absolute measure comparable across different queries. 3DPatch does not require the user to have prior knowledge of any 3D structures matching the query sequence, and will graphically present the coverage of the query sequence by the available 3D structures. Finally, it is important to note that 3DPatch is able to completely process typical queries in a matter of seconds, while the ConSurf server can take several tens of minutes to finish. Therefore, 3DPatch can be used as a part of a user's workflow without requiring them to shift focus away from their current task.

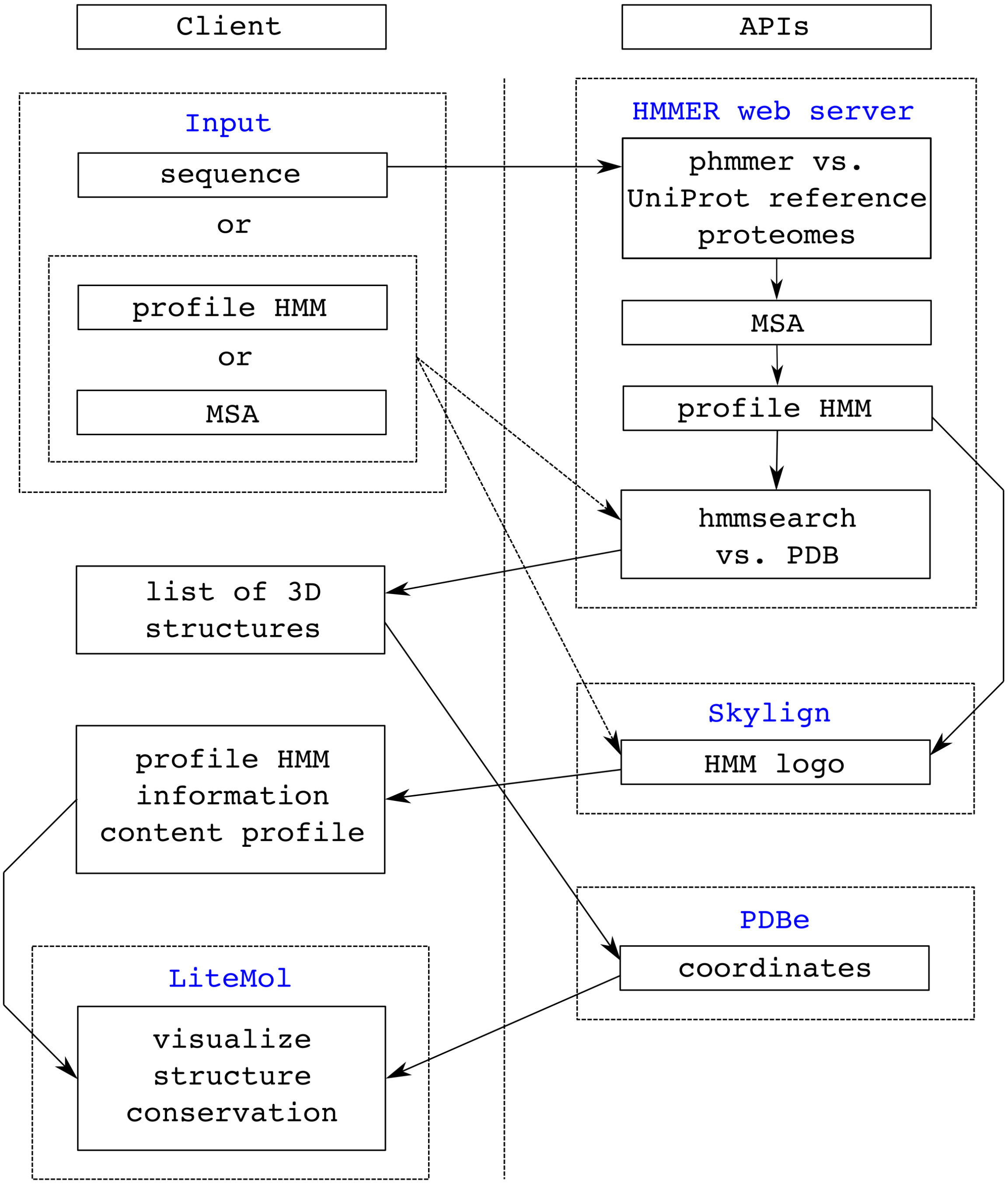

Supplementary Figure 1: 3DPatch workflow between the client and federated application programmatic interfaces.
